# Long Term Cardiovascular Outcome Based on Aspirin and Clopidogrel Responsiveness Status in Young ST-Elevated Myocardial Infarction Patients

**DOI:** 10.5935/abc.20180251

**Published:** 2019-02

**Authors:** Mustafa Umut Somuncu, Ali Riza Demir, Seda Tukenmez Karakurt, Huseyin Karakurt, Turgut Karabag

**Affiliations:** 1 Bulent Ecevit University - Faculty of Medicine -, Department of Cardiology, Zonguldak - Turkey; 2 Istanbul Mehmet Akif Ersoy Thoracic and Cardiovascular Surgery Training and Research Hospital, Istanbul - Turkey; 3 Istanbul University, Istanbul - Turkey

**Keywords:** Acute Coronary Syndrome, Aspirin/adverse effects, Platelet Aggregation, Young Adult, ST Elevation Myocardial Infarction, Mortality

## Abstract

**Background:**

A subset of patients who take antiplatelet therapy continues to have recurrent cardiovascular events which may be due to antiplatelet resistance. The effect of low response to aspirin or clopidogrel on prognosis was examined in different patient populations.

**Objective:**

We aimed to investigate the prevalence of poor response to dual antiplatelet therapy and its relationship with major adverse cardiovascular events (MACE) in young patients with ST-elevation myocardial infarction (STEMI).

**Methods:**

In our study, we included 123 patients under the age of 45 with STEMI who underwent primary percutaneous intervention. A screening procedure to determine both aspirin and clopidogrel responsiveness was performed on the fifth day of admission. We followed a 2x2 factorial design and patients were allocated to one of four groups, according to the presence of aspirin and/or clopidogrel resistance. Patients were followed for a three-year period. A p-value less than 0.05 was considered statistically significant.

**Results:**

We identified 48% of resistance against one or more antiplatelet in young patients with STEMI. More MACE was observed in patients with poor response to dual platelet therapy or to clopidogrel compared those with adequate response to the dual therapy (OR: 1.875, 1.144-3.073, p < 0.001; OR: 1.198, 0.957-1.499, p = 0.036, respectively). After adjustment for potential confounders, we found that poor responders to dual therapy had 3.3 times increased odds for three-year MACE than those with adequate response to the dual therapy.

**Conclusion:**

Attention should be paid to dual antiplatelet therapy in terms of increased risk for cardiovascular adverse events especially in young patients with STEMI.

## Introduction

Acute coronary syndrome (ACS) is considered to be the most important cause of death throughout the world, especially in western countries, despite technological improvements, new drugs and an increasing level of awareness.^1^ It has been found that aspirin therapy inhibits cardiovascular and cerebrovascular disease in approximately one out of every four patients.^2^ In patients with coronary artery disease, antiplatelet therapy has been included as a Class 1 recommendation in European guidelines.^3^ ischemic events continue to occur in a significant proportion of patients on antiplatelet therapy. This can be related to increased platelet activity resulting from the use of these drugs, which is called antiplatelet resistance.

Increasing evidence suggests that antiplatelet resistance occurs in varying rates in patients who are at risk for atherothrombotic complications. Moreover, the effect of biochemically detected antiplatelet resistance on cardiovascular adverse events has been found in different studies.^4-6^ In a meta-analysis with 50-plus studies, the association of aspirin and clopidogrel resistance with cardiovascular events was clearly indicated.^7^

Despite the use of more potent antiplatelets such as ticagrelor and prasugrel, clopidogrel continues to be used in a significant number of patients, sometimes due to financial constraints, and sometimes because of the risk of bleeding. Aspirin and clopidogrel resistance may lead to serious consequences especially in younger myocardial infarction (MI) patients because of the lifelong use. Low response to aspirin and clopidogrel has been studied separately in different groups of patients and its influencing factors have been investigated several times. However, there is insufficient data about both aspirin and clopidogrel response together. In addition, as far as we see, all studies evaluated the prevalence and prognostic effect of the dual antiplatelet resistance on young MI patients. Thus, in our study, the prevalence of aspirin and clopidogrel resistance and the relationship of low response to dual antiplatelet therapy with major adverse cardiovascular events (MACE) was assessed in young ST-segment elevation myocardial infarction (STEMI) patients who underwent primary percutaneous coronary intervention (PCI). Thus, we aimed to measure the prevalence of dual antiplatelet resistance in younger MI patients and to evaluate the effects of such poor response on their medical condition.

## Methods

### Patient population

In this prospective observational study, 123 consecutive patients (< 45 years old), who were admitted to a large-volume center with a diagnosis of STEMI and underwent primary PCI were included in the study. The exclusion criteria were: previous treatment with glycoprotein IIb/IIIa inhibitors, anticoagulant or non-steroid anti-inflammatory drugs in the last ten days, active malignancy, chronic inflammatory conditions, hemorrhagic diathesis, thrombolytic treatment within the last month, severe renal or liver disease and platelet counts < 100,000/mL, hematocrit count < 30% and no indication or unsuccessful of PCI. STEMI patients were defined as patients with typical chest pain at rest lasting more than 30 minutes, and ST-segment elevation ≥ 0.2 mV in 2 or more contiguous, precordial leads or adjacent limb leads on the standard 12-lead electrocardiogram (ECG). All primary PCI procedures were performed by operators who perform more than 100 PCIs/year at a single center (> 3000 PCIs/year). The minimum number of patients needed to be included for an effect size of 0.4 and 80% power was 156 for independent samples t-test and Mann-Whitney U test. During the follow-up 33 patients were excluded from the study due to suspected use of medications and finally, 123 patients were included in the study. The power for the final sample size was calculated at 70%. Sample size was calculated using the G-Power 3.9.1.2 package program and was also valid for other statistical tests used in the study. Initially, patients would be allocated into 2 groups - patients with drug resistance (n = 59) and drug responders (n = 64). However, to in order to make randomization between the groups more precise, 4 groups were formed according to the response to the drugs combined or alone.

The study complied with the Declaration of Helsinki. Written informed consent was obtained from all patients who participated in the study and the study protocol was approved by the ethics committee of our university.

### Analysis of patient data

Patients’ demographic data, past medical history, and previous medical therapies were collected. Risk factors were categorized as having or not having STEMI. Twelve-lead ECG was recorded for each patient immediately after hospital admission and the MI type was defined from the ECG. At 24-72 h after revascularization, a transthoracic echocardiography (Vivid S5 probe 3 S-RS/GE Healthcare, Wauwatosa, Wisconsin, USA) was performed to calculate left ventricular ejection fraction (LVEF) by using the biplane Simpson method.^8^ Primary angioplasty was performed only for infarct-related artery (IRA) occlusion (either total or partial). Intervention success was defined as reduction of IRA obstruction or stenosis to 30%, with TIMI 3 flow just after coronary intervention.

### Study design

 In this prospective observational study, we followed a 2x2 factorial design to create groups according to the presence of aspirin and clopidogrel resistance; poor responders to aspirin (n = 20, 39.7 ± 3.7 years old), poor responders to clopidogrel (n = 23, 39.6 ± 4.1 years old), dual poor responders (n = 16, 40.5 ± 4.1 years old), dual responders (n = 64, 38.7 ± 4.0 years old). All patients received dual antiplatelet therapy for 1 year after discharge. After one year, aspirin was prescribed with cardiac therapy. Patients were called for control at the first month after the procedure, and every six months thereafter, and the compliance was checked. Patients who did not use antiplatelet therapy in the follow-up period were excluded from the study. At the end of three years, patients were asked about the occurrence of cardiovascular events and the relationship between these events and the response to antiplatelet agents was evaluated.

### Evaluation of antiplatelet resistance

All participants received a chewable 300 mg or 100 mg aspirin (according to previous usage) and clopidogrel (600 mg loading dosage) before coronary angiography. Heparin (100 IU/kg) was administered after the decision to perform coronary intervention. After angioplasty, all patients were admitted to the coronary care unit, where routine antithrombotic therapy was given as daily dose 100 mg of aspirin, 75 mg of clopidogrel and subcutaneous administration of enoxaparin. The timing of platelet aggregation tests to identify hyporesponsiveness is also important. Thus, a screening procedure to determine aspirin and clopidogrel responsiveness was performed on the fifth day of admission to facilitate the steady state of drugs to be sure that platelet aggregation test was performed when maximal inhibition had been achieved. Whole blood aggregation was carried out with an impedance aggregometer, a Multiplate® platelet function analyser that operates on the surface of activated platelets to activate receptors that allow them to bind to artificial surfaces (Multiplate®; Dynabarte GmbH, Munich, Germany). Platelet aggregation was quantified as area under the curve, aggregation degree, and aggregation velocity. Platelet aggregation results were presented as aggregation unit (AU) × min, and values over 500 AU × min were defined as resistance to antiplatelet agents (used in combination or separately).^9^

### Follow-up

Patients’ data during follow-up were obtained from hospital records or by interviewing (in person or by telephone) the patients, their families, or their physicians. Primary clinical outcomes were composed of cardiovascular (CV) mortality, target vessel revascularization (TVR), non-fatal reinfarction, advanced heart failure and stroke. Secondary clinical outcomes were CV mortality, TVR, non-fatal reinfarction, stroke and advanced heart failure one by one.

### Statistical analysis

Statistical analysis was performed using the SPSS software version 18.0 for Windows (SPSS Inc., Chicago, Illinois, USA). Visual (histograms, probability plots) and analytical methods (Kolmogorov-Smirnov test, Shapiro-Wilk’s test) were used to assess the normal distribution of the variables. Descriptive analyses are presented as means and standard deviations for variables with normal distribution, as median and interquartile range for non-normal distribution. The categorical variables are expressed as numbers and percentages. Comparisons between the groups were performed using unpaired Student’s *t*-test or one-way ANOVA for continuous variables with normal distribution, and Kruskal-Wallis or Mann-Whitney *U* test for continuous variables without normal distribution. Tukey and Tamhane’s T2 tests were used based on the equal variance assumption in binary comparisons in groups with normal distribution and more than two independent variables. Mann-Whitney U test was used for the binary comparison of multiple groups with non-normal distribution. A Bonferroni correction was employed to adjust for multiple comparisons. Categorical data were compared with the chi-square test. Because of the statistical difference in the total model, the chi-square test was applied in binary groups to compare 3-year MACE results. The cumulative survival curve for 3-year cardiac mortality was executed using the Kaplan-Meier method, with differences assessed by log-rank tests. Multivariate Cox regression backward stepwise, that included variables with p < 0.01 on univariate analysis, was carried out to identify independent predictors of 3-year MACE. A p value less than 0.05 was considered statistically significant.

## Results

Among the 123 patients included in the study, the prevalence of poor responders to aspirin was 16.2%, to clopidogrel 18.6%, and to dual therapy 13.0%. In other words, in young MI patients, 47.8% of resistance against one or more antiplatelet was detected. Among baseline characteristics, hyperlipidemia, presence of family history, platelet counts, and platelet aggregation were different between the groups; no other differences were detected (Table 1).

**Table 1:** Baseline characteristics of the study population, mean ± standard deviation/median-interquartile range or n (%)π

	Adequate response to dual therapy (n = 64)	Poor response to aspirin (n = 20)	Poor response to clopidogrel (n = 23)	Poor response to dual therapy (n = 16)	p
Age, years ^β^	38.7 ± 4.0	39.7 ± 3.7	39.6 ± 4.1	40.5 ± 4.7	0.372
Male, n (%)	59 (92.2)	18 (90.0)	20 (87)	16 (100.0)	0.520
BMI, kg/m^2^	29.9 ± 4.6	28.6 ± 3.1	29.8 ± 4.2	29.3 ± 4.0	0.668
Hyperlipidemia, n (%)	19 (29.7)	9 (45)	14 (60.9)	10 (62.5)	0.017
Hypertension, n (%)	23 (35.9)	6 (30)	6 (26.1)	6 (37.5)	0.810
Diabetes mellitus, n (%)	7 (10.9)	3 (15)	3 (13)	1 (6.3)	0.861
Smoking, n (%)	46 (71.9)	13 (65.0)	17 (73.9)	11 (68.8)	0.919
Family history, n (%)	4 (6.3)	4 (20)	6 (26.1)	6 (37.5)	0.008
Total Chol. mg/dL ^β^	185.8 ± 48.7	188.4 ± 40.0	200.5 ± 48.7	208.7 ± 42.3	0.277
HDL, mg/dL^β^	37.0 ± 11.8	36.4 ± 9.4	38.6 ± 7.9	34.3 ± 7.9	0.652
LDL, mg/dL^β^	122.2 ± 34.1	126.0 ± 31.9	142.6 ± 42.1	137.3 ± 37.6	0.104
Triglycerides, mg/dL^¥^	121.5(69.7-202.2)	111.5(83.0-207.2)	101.0(62.0-194.0)	174.0(142.0-264.0)	0.060
Creatinine, mg/dL^¥^	0.80(0.80-0.90)	0.80(0.70-0.90)	0.80(0.80-1.00)	0.90(0.80-0.97)	0.417
Hematocrit, %^β^	43.0 ± 4.0	44.8 ± 4.8	42.3 ± 5.2	44.3 ± 2.6	0.202
Platelet, 10^3^ µL* ^β^	256.5 ± 45.5	309.4 ± 71.2	300.2 ± 81.1	300.3 ± 77.5	0.001
LVEF, %^¥^	50.0(45.0-56.5)	50.0(42.7-55.0)	55.0(50.0-60.0)	51.5(41.2-58.7)	0.244
**Culprit artery, %**					**0.449**
LAD	33 (51.6)	11 (57.9)	11 (47.8)	9 (56.3)	
CX	9 (14.1)	6 (31.6)	5 (21.7)	3 (18.8)	
RCA	22 (34.4)	2 (10.5)	7 (30.4)	4 (25.0)	
Syntax Score	17.6 ± 9.0	19.4 ± 10.7	17.6 ± 7.4	16.3 ± 7.3	0.766
Aspirin aggregation time (AU x min) ^‡β^	277.0 ± 98.9	789.1 ± 203.0	300.7 ± 133.7	738.0 ± 191.2	< 0.001
Clopidogrel aggregation time (AU x min) ^¶¥^	288.5 ± 234.0-376.0)	347.0(280.2-407.2)	608.0(523.0-728.0)	685.0(607.2-766.0)	< 0.001

BMI: body-mass index; Chol: cholesterol; HDL: high-density lipoprotein; LDL: low-density lipoprotein; LVEF: left vetricular ejection fraction; LAD: left anterior descending artery; CX: circumflex artery; RCA: right coronary artery; AU: aggregation unit; min: minute.

* p values < 0.05, dual therapy responders vs. other groups;

‡ p value < 0.05, aspirin poor responders vs. adequate response to aspirin:

¶ p values < 0.05, clopidogrel poor responders vs. adequate response to clopidogrel

¥ Kruskal-Wallis test was used for multiple independent variables without normal distribution, and Mann-Whitney U test was used for binary comparisons;

π Categorical data were compared with a chi-square test. B One-way ANOVA test was used for multiple independent variables with normal distribution, and for post hoc analysis, Tamhane's T2 and Tukey test were used.

**Figure 1:**
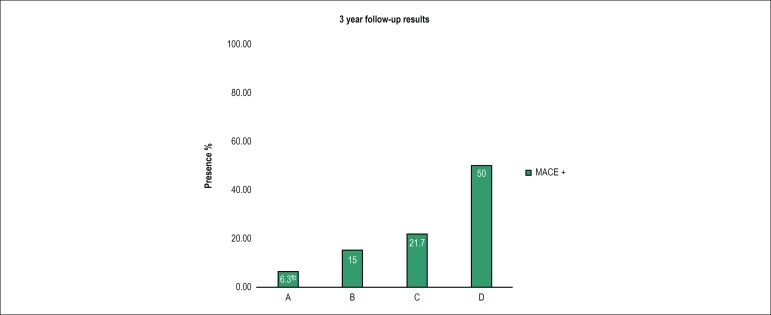
Bar graph showing major cardiovascular adverse events, in the four groups based on aspirin and clopidogrel response. (A) Patients with adequate response to dual antiplatelet therapy; (B) patients with low response to aspirin; (C) patients with low response to clopidogrel; (D) patients with low response to dual antiplatelet therapy. MACE: major adverse cardiovascular events. ^¶^ compared with poor responders to dual antiplatelet therapy (OR:1.875 1.144-3.073, p < 0.001); ^‡^ compared with patients with poor response to clopidogrel (OR: 1.198, 0.957-1.499, p = 0.036).

At the 3-year follow-up, the difference in the primary outcome (composed of CV mortality, non-fatal reinfarction, TVR, advanced heart failure, and stroke) was statistically significant between the groups (p < 0.001). When we analyzed secondary outcomes, cardiac mortality and TVR were statistically higher in the group of poor responders to dual therapy (p = 0.002, p = 0.010 respectively) (Table 2). More MACE was observed in the group of poor responders to dual therapy and clopidogrel poor responders compared to the group of dual responders (OR: 1.875, 1.144-3.073, p < 0.001; OR: 1.198, 0.957-1.499, p = 0.036, respectively) (Figure 1).

**Table 2:** Three-year outcomes of the study population, n (%)‡

Variable	Dual therapy responders (n = 64)	Aspirin poor responders (n = 20)	Clopidogrel poor responders (n = 23)	Poor responders to dual therapy (n = 16)	p
Primary outcomes *	4 (6.3)	3 (15.0)	5 (21.7)	8 (50.0)	< 0.001
**Secondary outcomes**^†^					
Cardiac mortality	0 (0)	1 (5.0)	0 (0)	3 (18.8)	0.002
Non-fatal MI	1 (1.6)	1 (5.0)	2 (8.7)	2 (12.5)	0.283
TVR	0 (0)	1 (5.0)	3 (13.0)	3 (18.8)	0.010
Stroke	0 (0)	0 (0)	0 (0)	0 (0)	---
Advanced heart failure	3 (4.7)	1 (5.0)	2 (8.7)	2 (12.5)	0.671

TVR: target vessel revascularization; MI: myocardial infarction; Asp: aspirin; Clop: Clopidogrel.

* Primary clinical outcomes were composed of cardiovascular (CV) mortality, non-fatal reinfarction, target vessel revascularization(TVR), advanced heart failure, stroke.

† Secondary clinical outcomes were CV mortality, non-fatal reinfarction, TVR, stroke, and advanced heart failure separately;

‡ all data in the table were compared byt the chi-square test and expressed as percentages.

**Figure 2:**
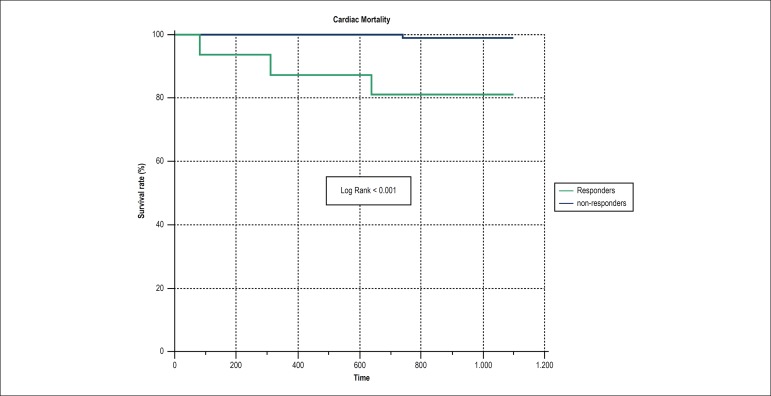
Kaplan-Meier analysis showing 3-year cardiac mortality rate according to antiplatelet response. Patients with adequate response to aspirin and/or clopidogrel were considered “responders”. Patients with both aspirin and clopidogrel resistance were considered non-responders.

In logistic regression analysis, family history, LVEF and clopidogrel aggregation time were identified as independent predictors of MACE in 3 years. Besides, we found that being a poor responder to dual therapy had 3.3 times increased odds for 3-year major adverse cardiovascular events than being in the dual responder group independent from family history and LVEF (Table 3). Moreover, the Kaplan-Meier survival plot for three-year CV mortality in dual poor responders and responders to one or both antiplatelet drugs is presented in Figure 2.

**Table 3:** Multivariate logistic regression analysis for potential predictors of major adverse carediovascular events at three-year follow-up

	Univariate analysis		Multivariate analysis	
	OR (95%CI)	p value	OR (95%CI)	p-value
**First Model^¶^**				
Age, years	1.079 (0.951- 1.225)	0.239		
Male	2.420 (0.569-10.292)	0.231		
Family history	5.056 (1.720-14.861)	0.003	5.972 (1.449-24.615)	0.013
Hyperlipidemia	1.142 (0.435-2.994)	0.788		
Diabetes mellitus	3.481 (1.026-11.810)	0.045	5.194 (0.884-30.540)	0.068
Hypertension	2.323 (0.878-6.142)	0.089	3.271 (0.823-12.998)	0.092
Culprit artery*	4.583 (1.434-14.650)	0.010	2.959 (0.604-14.498)	0.181
LVEF, %	0.878 (0.823-0.938)	< 0.001	0.832 (0.761-0.909)	< 0.001
Creatinine, mg/dl	0.828 (0.051-13.450)	0.894		
Asp agg. time (AU x min)	1.002 (1.000-1.003)	0.078	1.000 (0.998-1.003)	0.838
Clop agg time (Au x min)	1.002 (1.000-1.004)	0.041	1.003 (1.000-1.006)	0.022
**Second Model^†^**				
Responder^‡^	Ref.	Ref.	Ref.	Ref.
Asp res^‡^	2.647 (0.539-12.992)	0.230	2.075 (0.503-8.549)	0.312
Clop res^‡^	4.167 (1.011-17.175)	0.048	4.056 (0.618 -25.612)	0.065
Dual res^‡^	15.000 (3.666-61.366)	<0.001	3.334 (0.484-22.954)	0.002

CI: confidence interval; LVEF: left ventricular ejection fraction; Asp: aspirin; Clop: Clopidogrel; agg; aggregation; min: minute; AU: aggregation unit; res: resistant; MACE: major adverse cardiovascular events; OR: odds ratio.

* Culprit artery was divided as left anterior descending artery (LAD) and non-LAD (circumflex artery and right coronary artery);

‡ These groups were included in a second model instead of aspirin and clopidogrel aggregation time;

¶ Nagelkerke R square of the first model was 49.2%;

† Nagelkerke R square of the second model was 59.4%.

## Discussion

We can summarize the findings of our study as follows: (a) among STEMI patients under the age of 45 years who underwent PCI, 47.8% have a poor response to aspirin and/or clopidogrel; (b) poor responders to both aspirin and clopidogrel had a significantly higher level of MACE at 3 years follow-up compared with dual responders. Furthermore, secondary outcome analysis showed a significant difference in cardiac mortality and TVR between these two groups; (c) after adjustment for potential confounders, it was found that being a dual poor responder was one of the independent predictors of MACE. Moreover, the Kaplan-Meier survival plot for three-year CV mortality showed poor prognosis of dual poor responder patients (log rank < 0.001).

Antiplatelet resistance is a multifactorial phenomenon that has been studied in many populations with different methods. Therefore, the presence of variable results in the literature makes it difficult to compare our results with those of other studies. However, the lack of previous studies in young STEMI patients and long-term results of the dual antiplatelet resistance in this group make this study unique and valuable.

There is no single way to initiate thrombotic events; therefore, inhibition of a single pathway does not prevent all thrombotic complications. In addition, in some patients, the sensitivity of aspirin and clopidogrel is low, resulting in clinical complications. Therefore, several studies have been conducted to determine the clinical implications of being poor responders to aspirin and/or clopidogrel. In a meta-analysis of 1,813 patients with 12 studies examining the effect of aspirin resistance on prognosis, the mean biochemical aspirin resistance was 27% and the odds ratio for MACE was 3.8 (95% CI: 2.3-6.1) in patients with aspirin resistance.^4^ In another meta-analysis of 2,930 patients, aspirin resistance was detected in 28% of these patients, cardiovascular events in 41% (OR 3.85, 95% CI: 3.08-4.80), mortality in 5.7% (OR 5.99, 95% CI: 2.28-15.72) and acute coronary syndrome in 39.4% (OR 4.06, 95% CI: 2.96-5.56).^5^ In another study with patients with symptomatic peripheral artery disease, aspirin resistance was found as an independent predictor of adverse cardiovascular events with 2.48 hazard ratio.^10^ In a study on non-STEMI patients, aspirin resistants were at significantly higher risk of cardiovascular death with hazard ratio of 2.6 (95% CI 1.6-4.3) than aspirin sensitives (23.1% versus 9.6%).^11^ Although all of the above studies showed an association of aspirin resistance with cardiovascular events, in our study, the increase in MACE in poor responders to aspirin did not reach statistical significance (15% versus 6%, p = 0.217). These differences may be explained by several factors. Firstly, the lack of statistical significance may have been caused by our smaller sample size. Secondly, these studies were carried out in different groups of patients using different methods. Besides, in these studies above mentioned, there was no analysis of a subgroup of young patients. We may speculate that aspirin resistance in this group of patients may not affect cardiovascular events due to different pathophysiological mechanisms. However, synergistic contribution to the increase in cardiovascular events with clopidogrel responsiveness was detected in our study. Larger studies need to clarify this conflict.

When studies on clopidogrel response are reviewed, it can be seen that clopidogrel resistance is clinically expressed in different patient groups. In a meta-analysis investigating the ability of different platelet-function tests to reliably identify patients at risk of developing secondary cardiovascular events, Wisman et al.^7^ evaluated high on-aspirin and high on-clopidogrel platelet reactivity in 55 studies with 22,441 patients and in 59 studies with 34776 patients respectively. The high on-aspirin platelet reactivity rate was 22.2%, which was associated with an increased risk for cardiovascular events (relative risk [RR] 2.09; 95% confidence interval [CI] 1.77-2.47). They reported a high on-clopidogrel platelet reactivity in 40.4% of patients, which was associated with increased cardiovascular event risk (RR 2.80; 95% CI 2.40-3.27). Moreover, ten studies identified an increased cardiovascular event risk in patients with high-on dual platelet reactivity (RR 2.77; 95% CI 1.87-4.12). In our study, although patients resistant to either aspirin or clopidogrel showed more cardiovascular events, this was not statistically significant. This may be explained by our relatively small sample size. However, similar to the meta-analysis, poor response to dual therapy was found to be an independent predictor of MACE (RR 3.33; 95% CI 0.484-22.954). Furthermore, according to this meta-analysis,^7^ the Multiplate test, the same method used in our study, is one of the most reliable methods to identify cardiovascular events.

The effect of antiplatelet resistance on stent thrombosis as a clinical outcome was examined in some studies. Slottow et al.^12^ compared 26 patients who admitted with stent thrombosis under dual antiplatelet therapy with a control group to determine the relationship between stent thrombosis and antiplatelet resistance.^12^ In this study, aspirin and clopidogrel reaction units were significantly higher in patients with early drug-eluting stent thrombosis. Similar to these results, in two other studies evaluating clopidogrel resistance, stent thrombosis was seen more frequently after 6 months of follow-up.^13,14^ In a study comparing clopidogrel response with phenotyping and genotyping, patients with poor response to clopidogrel detected by multiple electrode aggregometry (MEA) had a higher risk of developing MACE or stent thrombosis than clopidogrel responders (12.5% vs. 0.3%, p < 0.001, and 18.5% vs. 11.3%, p = 0.022, respectively).^15^ Although we did not evaluate any stent thrombosis parameter, the frequency of cardiac mortality and TVR was significantly higher in patients with poor response to dual therapy than responders to dual therapy.

In the literature, we identified only one study with a similar grouping design, i.e., considering the response (responders vs. poor responders) to dual platelet therapy. Campo et al.^16^ evaluated the responsiveness status of aspirin and clopidogrel in 1,277 patients after elective PCI.^16^ In this study, at one-year follow-up they found that poor response to clopidogrel is an independent predictor of periprocedural MI and cardiovascular events whereas poor response to aspirin failed to predict a worse outcome. A distinctive feature of this study was that aspirin and clopidogrel response of 207 patients were evaluated together in subgroups and 25 patients were identified as the dual poor responder. In this subgroup analysis, the one-year composite endpoint of overall mortality, MI, and stroke was higher for dual poor responders compared with responders largely driven by a higher rate of MI (20% vs. 8.6%; p = 0.007). It may be expected lower cardiac mortality rates in our study group due to their younger age; however, our study had a longer follow-up than the above-mentioned study, which may have compensated for this. As a result, similar to the above study, we found a significant difference between the groups of nonresponders and the responders in terms of cardiac mortality (18.8% vs. 5.0%, p = 0.002).

There are also studies showing that platelet function tests do not have a prognostic significance in contrast to our results. Reny et al.^17^ detected that neither specific nor aggregation-based assays of antiplatelet drug responsiveness have additional predictive contribution to the recurrence of ischemic events in stable cardiovascular patients.^17^ But in this study, patients who had acute ischemic events less than one month before inclusion were excluded from the study. Consequently, poor antiplatelet drug response may be less critical in a stable cardiovascular patient because of less endothelial thrombogenicity and less platelet activation in the stable patients shown in previous studies.^18-20^ It may be assumed that platelet function tests may have more impact on clinical outcomes in our study group when considering that platelet activation is related to inflammatory processes, and that inflammation is one of the most important factors in acute coronary syndromes, especially in young STEMI patients.

This study supports the view that standardized maintenance doses of antiplatelet drugs would not prevent MACE in some of the patients. Could it be possible to overcome platelet resistance by increasing the dose of medicine in our patient group? In some trials, increasing the dose of aspirin has allowed some reduction in aspirin resistance rates, but such effect is absent in 5-10% of patients. In addition, gastrointestinal hemorrhage and other side effects may increase when aspirin dose is increased in these patients. In addition, high doses of Aspirin can reduce the production of prostacyclin, an important endogenous vasodilator and antiplatelet agent, by inhibiting cyclooxygenase 2. Also, in our study, patients with only aspirin resistance did not differ in terms of MACE compared with patients with response to dual therapy, whereas patients with only clopidogrel resistance showed a significant difference. Geisler and colleagues have also shown that the response to clopidogrel may be reduced after acute coronary syndrome.^21^ This suggests that high platelet activity following acute coronary syndrome may be present and the standard dose of clopidogrel may not be sufficient to inhibit platelets. In parallel to this, it was found that administration of a 600 mg loading dose of clopidogrel in patients already chronically treated with clopidogrel provide additional inhibition of ADP-induced platelet aggregation.^22^ This information may be reflected in clinical practice, especially in some risky situations. Thus, in cases of inadequate response to clopidogrel, dose escalation or more potent inhibitors (ticagrelor, prasugrel) may be considered. For these reasons, whether high dose of aspirin or clopidogrel is beneficial to young MI patients with antiplatelet resistance is open to investigation.

There are some limitations in our study. First, this was a single-center study which may result in selection bias. Moreover, since we studied a specific population, the number of patients participating in the study was relatively small. This may have prevented the difference between some groups from reaching statistical significance. Second, antiplatelet sensitivity was only measured once, and some researchers have suggested that it should be measured more than once. Furthermore, when heterogeneous results of different studies are considered, the use of a single laboratory method constitutes one important limitation of the study. However, the multiple platelet function test reduces the risk of laboratory errors because it is faster, less troublesome, and does not require specific preparation than conventional optical aggregometry. Third, because of the study design, results of platelet sensitivity test cannot be generalized to different age groups with other forms of coronary artery disease. Fourth, clopidogrel was used as the second antiplatelet agent for STEMI, as the use of other P2Y12 inhibitors had not been included in the guidelines during the study period. Therefore, we do not know whether the use of more potent P2Y12 inhibitors would be associated with a lower prevalence of poor aspirin responders. Finally, aspirin and clopidogrel serum levels were not measured. However, the medical history of each patient was taken by one-to-one interview, and patients with irregular drug usage were excluded from the study.

## Conclusion

Although there are many studies in the literature on platelet response to different antiplatelet medications, many questions remain unanswered. In summary, we found that poor responsiveness to dual therapy is an essential predictor of MACE, including CV mortality and TVR in a three-year follow-up period in young patients undergoing primary PCI for STEMI. Although more potent P2Y12 inhibitors have been shown to be useful after acute coronary syndrome according to guidelines, there is no clear study of their use after one year. Therefore, aspirin or clopidogrel should be used in the long term after acute coronary syndrome, particularly in young MI patients, who may be more likely to antiplatelet resistance in long-term. For this reason, although routine testing for antiplatelet resistance is not recommended in the general population, it should be considered for young MI patients and, if resistance is detected, more potent antiplatelet therapy may be used one year after acute coronary syndrome. More comprehensive investigations are required to clarify this.
